# Successful Endoscopic Treatment of Walled‐Off Necrosis With Complex Quadruple Fistulas Among the Necrotic Cavity, Stomach, Duodenum, and Common Bile Duct

**DOI:** 10.1002/deo2.70432

**Published:** 2026-09-11

**Authors:** Shuhei Shibata, Yotaro Iino, Jo Kurosawa, Ken Uchida, Daiki Takahashi, Yuya Yokoyama, Hiroshi Ohyama, Futoshi Hirai, Michikazu Abe, Jun Kato

**Affiliations:** ^1^ Department of Gastroenterology Chiba Prefecture Saiseikai Narashino Hospital Chiba Japan; ^2^ Department of Gastroenterology Chiba University Hospital Chiba Japan

**Keywords:** endoscopy, fistula, pancreatitis, stents, walled‐off necrosis

## Abstract

We report an extremely rare case of duodenal bulb perforation caused by a double‐pigtail plastic stent placed for walled‐off necrosis (WON), coexisting with a biliary fistula to the WON and a choledochoduodenal fistula, all successfully managed with endoscopic treatment alone. A 61‐year‐old woman developed severe gallstone pancreatitis and underwent endoscopic ultrasound‐guided transmural drainage for WON with placement of a lumen‐apposing metal stent and a double‐pigtail plastic stent. After repeated endoscopic necrosectomy, the metal stent was removed and the plastic stent remained in place. Contrast leakage from the WON into the bile duct was detected, and a biliary stent was inadvertently deployed through a choledochoduodenal fistula. Two hundred thirty‐seven days after plastic stent placement, endoscopy revealed a duodenal bulb perforation with purulent discharge. The plastic stent was removed, and the gastric fistula was closed using endoscopic hand‐suturing. After spontaneous closure of the stent‐induced duodenal perforation was confirmed, the biliary stent placed through the choledochoduodenal fistula was removed and a new stent was inserted through the papilla of Vater, resulting in closure of the choledochoduodenal fistula. Complete closure of all fistulas was confirmed 516 days after the initial hospitalization. This case demonstrates that complex fistulas associated with WON can be successfully treated without surgical intervention.

## Graphical abstract

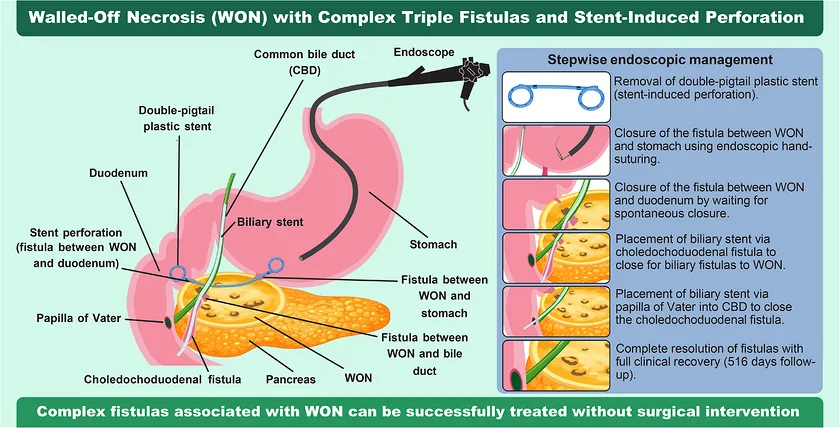

## 1 Introduction

Endoscopic ultrasound‐guided transmural drainage (EUS‐TD) has become widely accepted for walled‐off necrosis (WON) following acute pancreatitis. To prevent adverse events associated with lumen‐apposing metal stents, a double‐pigtail plastic stent is often inserted and frequently left in place even after removal of a lumen‐apposing metal stent. However, there is still no consensus regarding the optimal device type and duration of stent placement. Duodenal perforation caused by a double‐pigtail plastic stent is extremely rare, with only two previously reported cases [1, 2]. In addition, there has been only one previously reported case of a biliary fistula to a WON concomitant with a choledochoduodenal fistula [3].

We report a unique case in which these complex fistulous complications coexisted and were successfully managed endoscopically.

## 2 Case Report

A 61‐year‐old woman was admitted to our hospital with acute‐onset vomiting and abdominal pain and was diagnosed with severe acute pancreatitis. Apart from obesity, she had no relevant comorbidities, including immunodeficiency, diabetes mellitus, or malignancy, and no history of steroid or other immunosuppressive therapy. Gallstone pancreatitis was diagnosed retrospectively based on gallbladder stones and cholangitis, with no other identifiable cause. Although no common bile duct stone or stone impacted at the papilla was detected on imaging, spontaneous passage of a causative bile duct stone was suspected. Given the absence of persistent biliary obstruction that would necessitate urgent drainage, endoscopic intervention targeting potential choledocholithiasis was withheld. After intensive care, her condition improved; however, WON developed, and its progression resulted in duodenal stenosis, leading to impaired oral intake. EUS‐TD was performed, and both a lumen‐apposing metal stent and a double‐pigtail plastic stent were placed transgastrically into the WON. After five sessions of endoscopic necrosectomy, the WON cavity markedly decreased, and the lumen‐apposing metal stent was removed, whereas the double‐pigtail plastic stent was retained for internal drainage. Cholecystectomy was subsequently performed after her condition stabilized.

**FIGURE 1:**
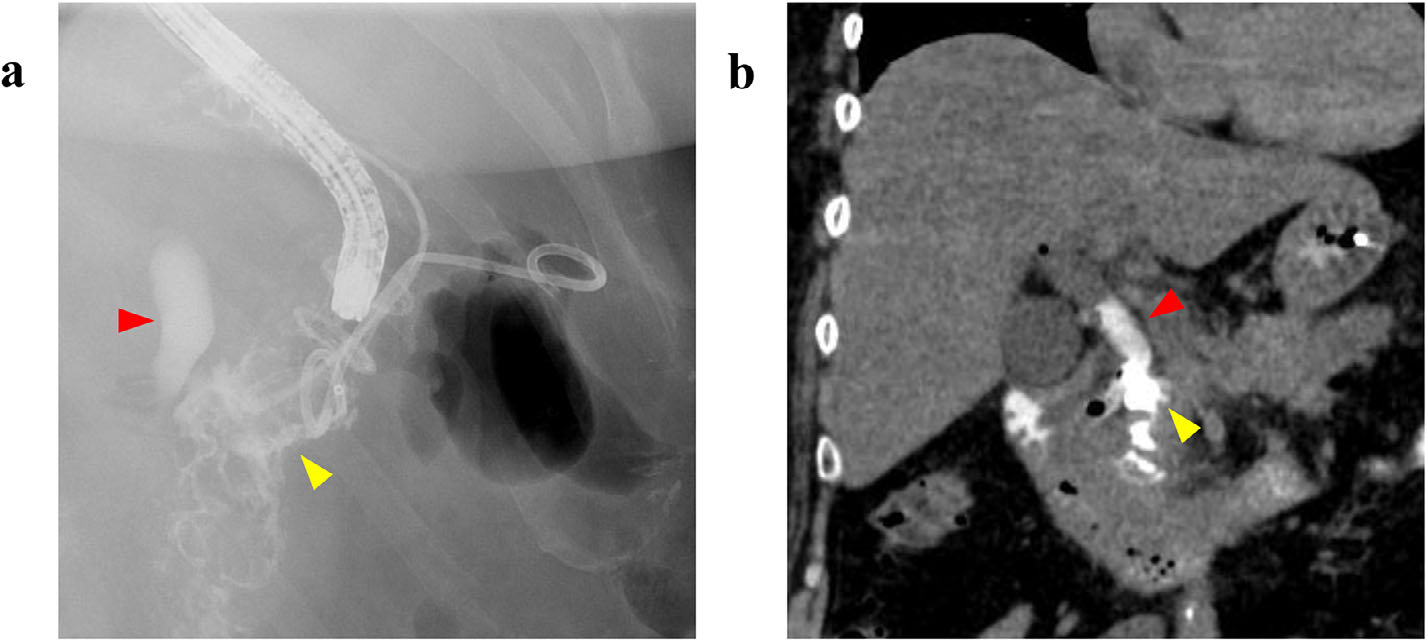
Fistula between the WON and the bile duct. Contrast medium was injected into the WON under endoscopic guidance. The red arrowhead indicates the bile duct, and the yellow arrowhead indicates the WON: (a) fluoroscopic image and (b) plain CT image. CT, computed tomography; WON, walled‐off necrosis.

During the final necrosectomy session, contrast medium leakage from the WON into the bile duct was observed. This finding necessitated subsequent ERCP. The duodenal mucosa was severely edematous due to inflammatory involvement, and the papilla of Vater could not be clearly identified. Instead, pus was observed draining from an opening in the duodenum, which was presumed to be the papilla of Vater. Cannulation was performed at this site, and cholangiography confirmed the presence of a fistula between the WON and the bile duct (Figure 1). To promote fistula closure, a biliary stent was placed at the presumed papilla, but it was later determined that the stent had been deployed into a choledochoduodenal fistula. The patient's clinical condition improved with conservative management, and she was discharged on hospital Day 87.

**FIGURE 2:**
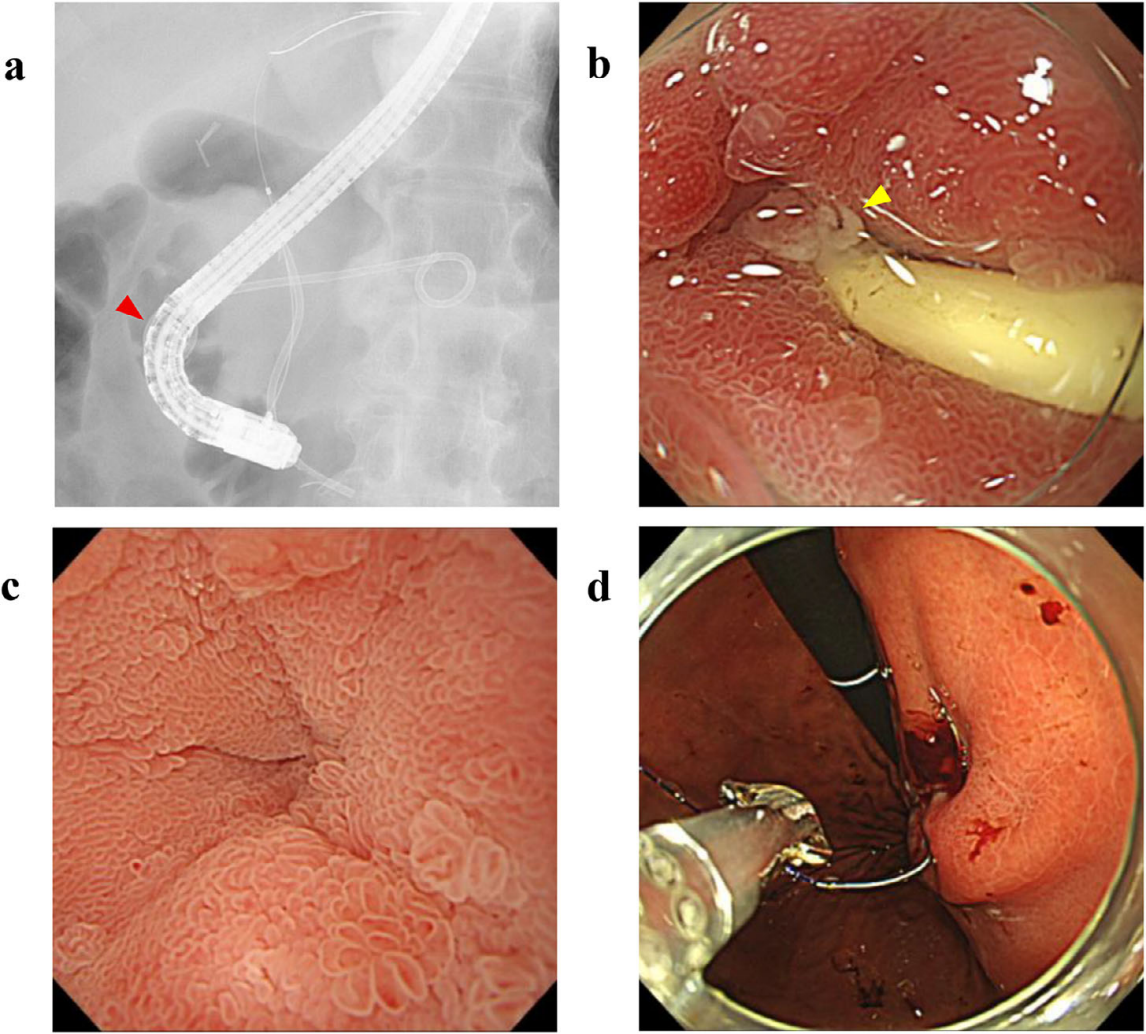
Duodenal bulb perforation caused by a double‐pigtail plastic stent placed for WON and its endoscopic management. (a) Fluoroscopic image. The red arrowhead indicates the double‐pigtail plastic stent, which is located behind the endoscope passing through the duodenum, suggesting perforation of the duodenal bulb. (b) Endoscopic image of the duodenal bulb perforation. The yellow arrowhead indicates purulent discharge. (c) Endoscopic image after stent removal, showing scarring. (d) EHS using SutuArt performed to close the gastric‐side fistula after stent removal. EHS, endoscopic hand‐suturing; WON, walled‐off necrosis.

**FIGURE 3:**
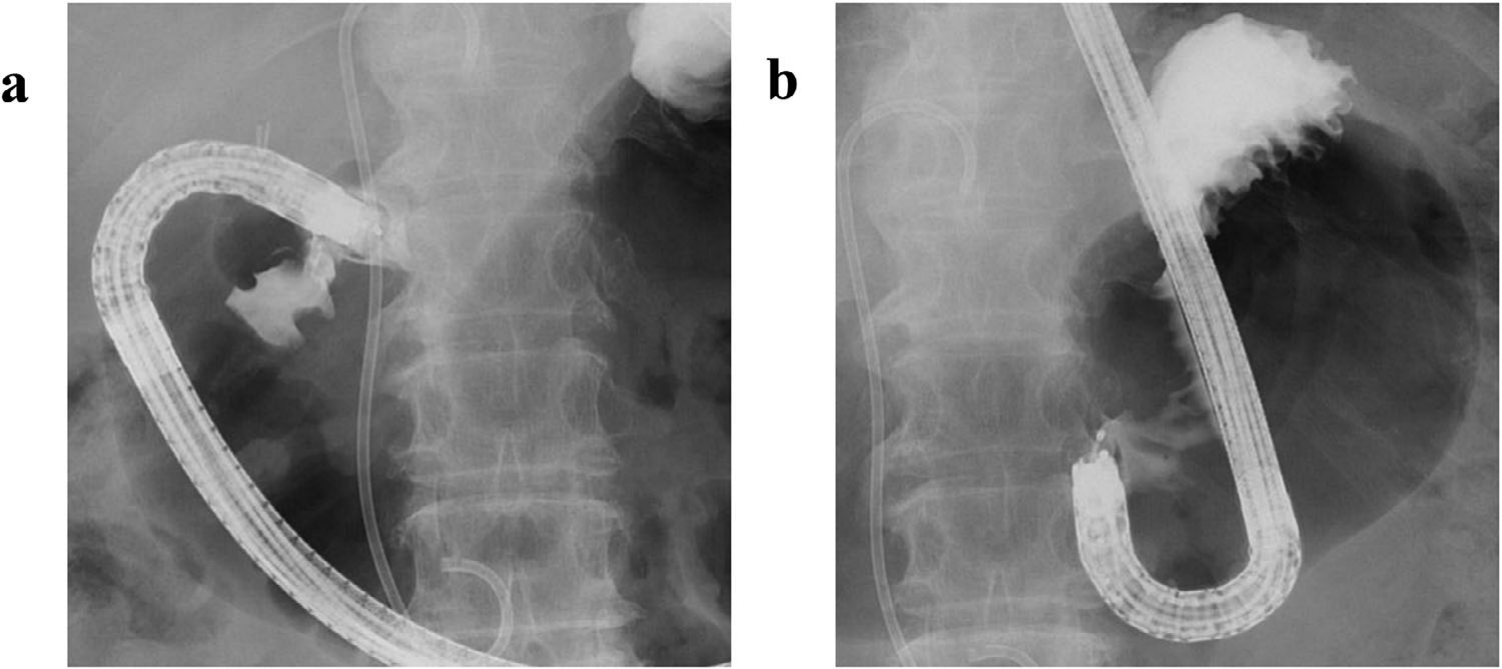
Contrast study after fistula closure: (a) duodenal bulb side and (b) gastric side.

The patient remained asymptomatic, with no abdominal pain. During this period, regular surveillance was performed using computed tomography (CT), magnetic resonance cholangiopancreatography (MRCP), and endoscopy. Because a residual WON cavity persisted and disconnected pancreatic duct syndrome was present (Figure S1), the double‐pigtail plastic stent was left in place to maintain internal drainage. However, follow‐up upper gastrointestinal endoscopy performed 237 days after placement of the double‐pigtail plastic stent revealed the perforation of the duodenal bulb by the stent (Figure 2a), with purulent discharge observed from both stent ends (Figure 2b). At that time, the WON was found to communicate with the stomach, duodenal bulb, and bile duct. The double‐pigtail plastic stent was removed (Figure 2c), and the gastric fistula was closed using endoscopic hand‐suturing (EHS) with SutuArt (Figure 2d). Oral intake was resumed 2 days after fistula closure. On Day 103 after the closure procedure, a contrast study performed through the closure site and the perforated area of the duodenal bulb demonstrated no extravasation of contrast medium outside the gastrointestinal tract (Figure 3).

Subsequently, ERCP was performed to confirm closure of the fistula between the WON and the bile duct. ERCP revealed that the previously placed biliary stent had not been deployed through the papilla of Vater but rather within a choledochoduodenal fistula. Closure of the biliary fistula to the WON was confirmed, and the stent within the choledochoduodenal fistula was removed.

**FIGURE 4:**
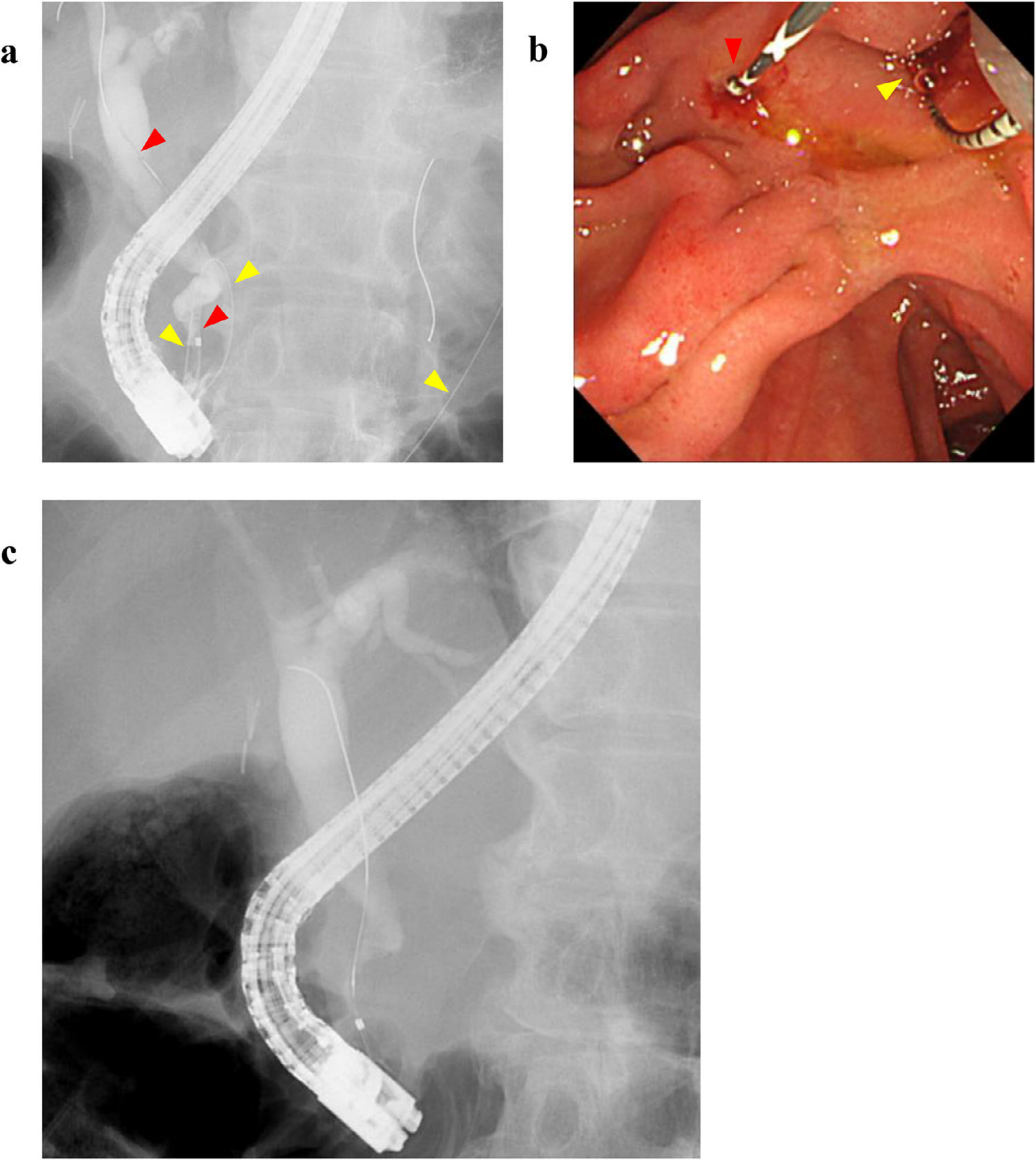
Endoscopic management of the fistula between the WON and the bile duct and the choledochoduodenal fistula. The red arrowhead indicates the guidewire inserted through the papilla of Vater, and the yellow arrowhead indicates the guidewire inserted through the choledochoduodenal fistula. (a) Fluoroscopic image. (b) Endoscopic image after cannulation through the papilla of Vater. Guidewires are placed in both the papilla of Vater and the choledochoduodenal fistula. (c) Fluoroscopic image showing the closure of both the biliary fistula to the WON and the choledochoduodenal fistula.

Due to the difficulty of cannulation through the papilla of Vater, a guidewire was instead advanced from the choledochoduodenal fistula into the bile duct and guided toward the papilla of Vater. Transpapillary cannulation was then successfully performed through the papilla along this guidewire, and an additional guidewire was advanced from the papilla into the common bile duct (Figure 4a,b). A new biliary stent was subsequently placed through the papilla of Vater.

A follow‐up ERCP performed 89 days later confirmed complete closure of the choledochoduodenal fistula (Figure 4c). Thus, all fistulas were confirmed to have closed 516 days after the initial hospitalization for severe gallstone pancreatitis. The patient is currently stent‐free and remains under outpatient follow‐up.

## 3 Discussion

Unlike previous cases managed by stent removal and fasting [1, 2], purulent discharge in our patient suggested residual WON infection. CT showed no free intraperitoneal air, supporting a contained duodenal communication rather than free perforation. Clip closure of both the gastric and duodenal openings failed because of mucosal edema. Because the stomach provided adequate working space for EHS, whereas the duodenal bulb did not, we closed only the gastric fistula to prevent inflow of food and digestive fluids while preserving the duodenal tract for drainage. Follow‐up imaging showed progressive reduction of the WON without worsening infection. The duodenal perforation likely resulted from stent displacement during WON shrinkage and subsequent pressure necrosis. Given the contained nature of the perforation and the potential drainage function of the duodenal tract, we elected to allow the duodenal perforation to close spontaneously.

Although the WON cavity gradually decreased, complete resolution was not achieved, resulting in a prolonged stent indwelling period of 237 days. Despite duodenal penetration, the patient remained asymptomatic, and regular surveillance with CT and endoscopy enabled detection of the stent‐related perforation. Earlier stent removal might have prevented this complication; however, purulent discharge from both stent ends suggested persistent infection, and earlier removal might have compromised internal drainage. Thus, the optimal timing of stent removal remained uncertain. Regular surveillance may therefore be important for early detection of stent‐related adverse events and timely reconsideration of continued stent placement.

There is no established treatment strategy for biliary fistulas to a WON or choledochoduodenal fistula. Surgical management has traditionally been recommended [4]. However, recent reports have demonstrated successful endoscopic closure of such fistulas [5, 6]. In our case, difficulty identifying the papilla during the initial ERCP led to inadvertent biliary stent placement through the choledochoduodenal fistula. The subsequent closure of the biliary fistula to the WON was likely attributable to biliary decompression rather than to the choledochoduodenal route itself. If technically feasible, transpapillary biliary drainage would have been more physiological and might have promoted earlier fistula closure. Thus, stenting through the choledochoduodenal fistula should be regarded as an unintended alternative route under technically challenging circumstances, rather than as a preferable drainage strategy. After closure of the biliary fistula to the WON was confirmed, a new biliary stent was placed through the papilla of Vater, followed by closure of the choledochoduodenal fistula.

This extremely rare case demonstrates that multiple complex fistulas associated with WON can be successfully closed without surgery through careful sequencing and stepwise endoscopic management.

## Author Contributions

Shuhei Shibata managed the case and drafted the manuscript. Yotaro Iino, Jo Kurosawa, Yuya Yokoyama, Futoshi Hirai, and Michikazu Abe provided clinical support and supervision and critically reviewed the manuscript. Ken Uchida, Daiki Takahashi, Hiroshi Ohyama, and Jun Kato contributed to manuscript review. All authors approved the final version of the manuscript.

## Funding

The authors have nothing to report.

## Ethics Statement

All procedures were conducted in accordance with the ethical principles outlined in the Declaration of Helsinki and its subsequent revisions.

## Conflicts of Interest

The authors declare no conflicts of interest.

## Supporting information




**Figure S1**: MRCP image of disconnected pancreatic duct syndrome.
**Figure S2**: CT images showing the clinical course of WON.

## Data Availability

The data that support the findings of this study are available from the corresponding author upon reasonable request.
