# Supplementary material for: Successful Endoscopic Treatment of Walled‐Off Necrosis With Complex Quadruple Fistulas Among the Necrotic Cavity, Stomach, Duodenum, and Common Bile Duct
Source: DEN Open. 2026 Sep 11;7(1):e70432. doi: 10.1002/deo2.70432 (PMC13569978; doi:10.1002/deo2.70432)
Supplement: Supplementary file 1 [file DEO2-7-e70432-s001.docx]

**Figure S1 MRCP image of disconnected pancreatic duct syndrome.**

Abbreviations: MRCP, magnetic resonance cholangiopancreatography

**Figure S2 CT images showing the clinical course of WON**

**a**

**b**

**c**

**d**

(a) At the time of WON diagnosis. (b) After EUS-TD. (c) At the time of stent-induced perforation. (d) After completion of treatment.

Abbreviations: CT, computed tomography; WON, walled-off necrosis; EUS-TD, endoscopic ultrasound-guided transmural drainage
